# Sutimlimab in Patients With Cold Agglutinin Disease (CAD): Results From a Managed Access Program

**DOI:** 10.1111/ejh.70068

**Published:** 2025-12-21

**Authors:** Sandra Maria Frey, Pauline Halberstadt, Ferras Alashkar, Veronika Lenz, H. Christian Reinhardt, Alexander Röth

**Affiliations:** ^1^ Department of Hematology and Stem Cell Transplantation University Hospital Essen, University of Duisburg‐Essen Essen Germany; ^2^ Institute of Transfusion Medicine University Hospital Essen, University of Duisburg‐Essen Essen Germany

**Keywords:** C1s inhibitor, cold agglutinin disease, long‐term follow‐up, sutimlimab

## Abstract

Cold agglutinin disease (CAD) is a low‐grade lymphoproliferative disorder accounting for 15%–30% of patients suffering from autoimmune hemolytic anemias. The clonal B cells produce autoantibodies primarily of the IgM‐κ class that cause agglutination of red blood cells (RBCs) at temperatures ≤ 37°C and activate the classical complement pathway with subsequent RBC destruction. Before the approval of the monoclonal antibody sutimlimab, treatment options were limited. Sutimlimab inhibits the classical complement pathway at C1s while sparing the lectin and alternative complement pathways. Efficacy, safety and tolerability of sutimlimab have been established in the multicenter CARDINAL and CADENZA trials. In the present investigation, 10 patients on sutimlimab were followed up to 96 weeks in the Managed Access Program (MAP) in Essen. Eight of these patients had previously participated in the multicenter trials and were restarted on sutimlimab after a median washout period of 12.6 weeks. In addition, two sutimlimab‐naive patients were included. Hemoglobin concentration increased in all patients (median hemoglobin concentration before therapy 9.8 vs. 12.5 g/dL at the end of the study). Bilirubin levels normalized already after 2 weeks in all patients. Reticulocyte count declined in nine, haptoglobin and hemopexin concentrations normalized in six and 10 patients, respectively. In conclusion, the present investigation with primary CAD patients in a single center demonstrates long‐term normalization of laboratory parameters, abrogation of specific clinical symptoms and absence of drug‐related side effects with sutimlimab.

## 1 Introduction

**FIGURE 1:**
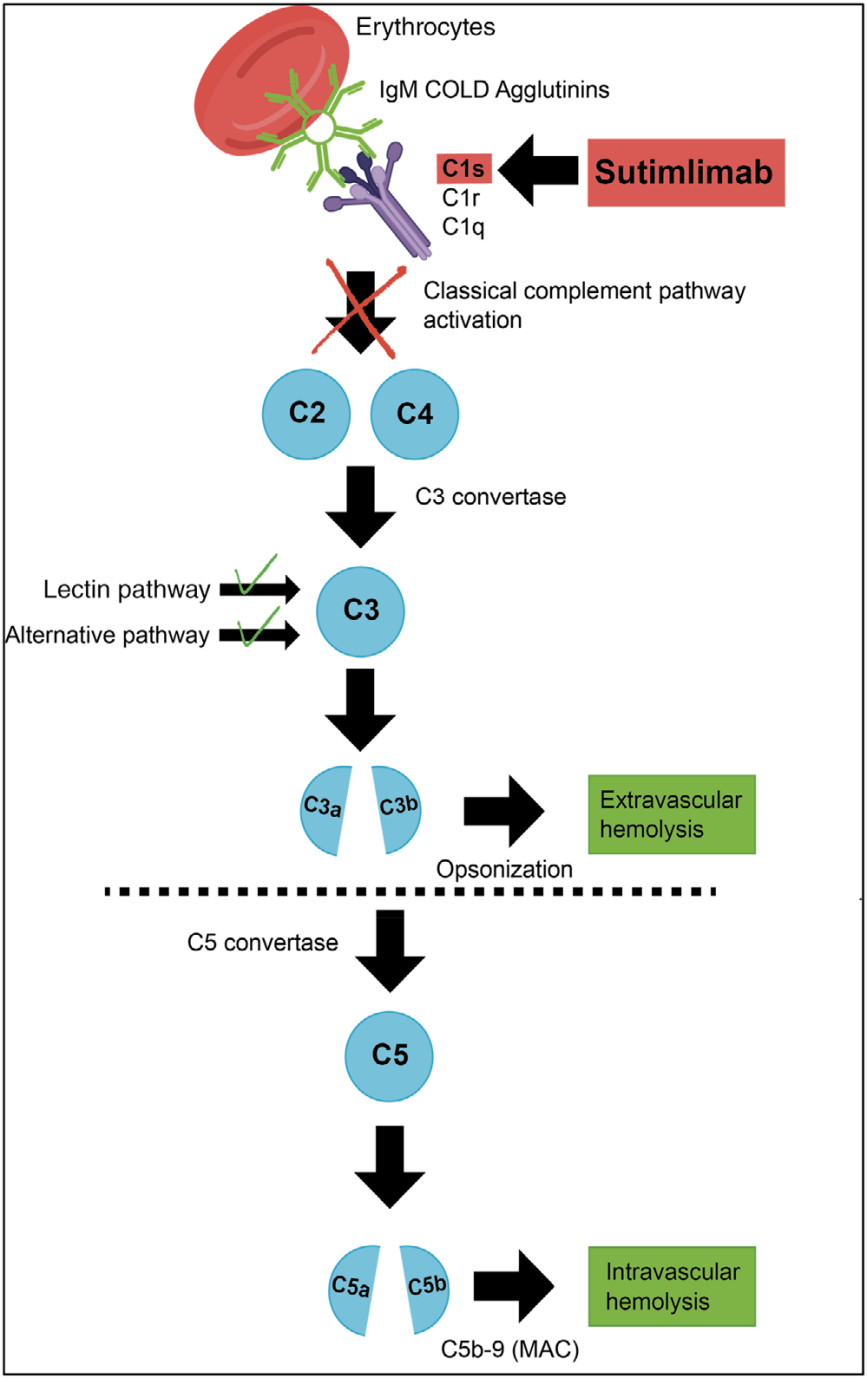
Classical complement pathway inhibition with sutimlimab. The lectin and alternative pathways remain unaffected.

Cold agglutinin disease (CAD) is a low‐grade lymphoproliferative disorder of clonal B cells that produce cold agglutinin, usually of the immunoglobulin M (IgM)‐κ class [1, 2, 3]. CAD is a member of the autoimmune hemolytic anemia family (AIHA) accounting for 15%–30% of all AIHA cases [1, 2, 4, 5]. As the name implies, the cold agglutinin can lead to agglutination of red blood cells (RBC) at ≤ 37°C after recognizing the “I” antigen on the surface of RBCs [5, 6]. The resulting cold agglutinin–antigen complexes on the RBC surface activate the classical complement pathway (CP) [7]. This activation by CP leads to RBC destruction, mainly by extravascular hemolysis and to a smaller extent by intravascular hemolysis mediated by the terminal complement pathway (Figure 1) [5, 6].

Patients with CAD exhibit a range of clinical manifestations, including complement‐mediated symptoms such as chronic hemolytic anemia and pronounced fatigue [6, 8]. In addition, they may experience cold‐induced agglutination‐mediated circulatory symptoms with acrocyanosis and Raynaud's phenomenon [5]. CAD is associated with an increased risk of thromboembolic events and early mortality [8, 9]. Due to these symptoms, patients with CAD often experience a reduced quality of life comparable with that of other patients with chronic diseases [5, 8].

Until recently, there was no approved therapy for CAD [4]. However, rituximab or chemoimmunotherapy with rituximab plus bendamustine was used in CAD patients and could induce even long‐lasting remissions in a proportion of cases. A few years ago, sutimlimab, a first‐in‐class humanized immunoglobulin monoclonal antibody that selectively inhibits C1s from the classical complement pathway, was approved based on two clinical trials, CARDINAL and CADENZA (Figure 1) [8, 10]. The purpose of the CARDINAL study was to evaluate the efficacy, safety and tolerability of sutimlimab in a prospective, open‐label, single‐arm, two‐part, multicenter phase 3 trial with recently transfused primary CAD patients [10]. The CADENZA trial was a randomized, placebo‐controlled, double‐blind phase 3 study with primary CAD patients without transfusions within 6 months prior to enrollment [8]. The duration of these two trials was restricted to 26 weeks with an open‐label extension of 1–2 years. Since the underlying lymphoproliferative disease in primary CAD patients is unaffected by sutimlimab, chronic treatment is necessary. Thus, long‐term efficacy and safety of sutimlimab are of relevance. Here we report eight patients who were treated with sutimlimab for an additional 96 weeks after completing the CARDINAL or the CADENZA trial after a washout period as well as two sutimlimab‐naive patients.

## 2 Methods

### 2.1 Study Design and Patients

The present retrospective analysis of 10 patients with symptomatic CAD, treated within the Managed Access Program (MAP) with sutimlimab, was conducted at the Department of Hematology and Stem Cell Transplantation of the University Hospital of Essen, Germany. All patients studied were Caucasians, older than 18 years, suffered from primary CAD and were treated at our institution. Primary CAD was defined as chronic hemolysis with a positive polyspecific direct antiglobulin test (DAT), a strongly positive monospecific DAT for C3d, a CA titer of ≥ 1:64 at 4°C and no evidence of malignancy [11].

All patients in the MAP were treated for 96 weeks with sutimlimab. Eight of 10 patients had participated in the CADENZA (*n* = 6) or CARDINAL (*n* = 2) trial [8, 12]. Both studies were conducted over a 26‐weeks treatment period, Part A, followed by an open‐label extension period, Part B [8, 12]. The duration of Part B of the CARDINAL study was 2 years and that of the CADENZA study was 1 year. After completion of Part B, the patients were observed without therapy for 9 weeks. Thereafter, the patients were included in the MAP. In addition to the eight patients participating previously in the CARDINAL or CADENZA trial, two naive patients were treated.

Sutimlimab was administered as in the CARDINAL or CADENZA trial by intravenous infusion over 60 min with a dose of 6.5 or 7.5 g, depending on whether the bodyweight was below or above 75 kg, on Day 0, Day 7, and every 14 days, thereafter. Patients were followed at two‐weekly intervals for a maximum of 96 weeks. A clinical assessment was performed in order to identify potential side effects of sutimlimab. The following laboratory measurements were obtained: hemoglobin, reticulocytes, LDH, total bilirubin, haptoglobin, hemopexin, complement C3 and C4.

### 2.2 Statistical Analysis

The range and the median were derived. Visual inspection of the data revealed the anticipated unequivocal effect of sutimlimab. Therefore, no formal statistical analysis was mandatory.

## 3 Results

### 3.1 Baseline Demographics

The CARDINAL study was conducted between March 2018 and October 2021 and the CADENZA trial from March 2018 until November 2021. Subsequently the MAP investigation was performed from November 2021 until October 2023. Ten patients with a symptomatic primary CAD were included in the MAP. Two patients were sutimlimab naive and eight patients were restarted on sutimlimab after a median washout period of 12.6 weeks following the CADENZA or CARDINAL trial. The observation period after the washout was 96 weeks and the total treatment period including the CARDINAL or CADENZA trial was up to 179 months. Patient characteristics are given in Table 1. Most patients were elderly females. All patients had laboratory evidence of hemolysis and CAD‐associated symptoms such as acrocyanosis, Raynaud's syndrome or hemoglobinuria. The monospecific Coombs test for C3d was analyzed in eight patients; thereof six patients had a strongly and two patients had a slightly positive result. Complement C4 was analyzed at irregular intervals. Before initiation of the therapy, the complement C4 measured in three patients was not detectable in one patient and in the normal range in two patients. At baseline, cold agglutinin antibodies were detectable in all 10 patients analyzed.

**TABLE 1:** Baseline characteristics.

Median (range), unless otherwise specified	Total (*n* = 10)
Age, years	70.5 (56–84)
Female sex, *n* (%)	8 (80)
Ethnicity: Caucasians, *n* (%)	10 (100)
Transfusions, *n* (%) in previous 6 month	2 (20)
CAD‐related disease characteristics, *n* (%)	
Acrocyanosis	6 (60)
Raynaud's syndrome	3 (30)
Hemoglobinuria	5 (50)
Disabling circulatory symptoms	4 (40)
Major adverse vascular event (including thrombosis), *n* (%)	1 (10)
History of hospitalization related to CAD within previous 2 years, *n* (%)	3 (30)
Laboratory results	
Hemoglobin, g/dL	9.8 (8.4–13.4)
Reticulocytes, 10^9^/L	136.5 (65.5–375.5)
LDH, U/L	392 (223–609)
Bilirubin mg/dL	2.0 (0.8–5.6)
Haptoglobin, g/L	0.01 (0.01–0.27)

### 3.2 Follow‐Up

**FIGURE 2:**
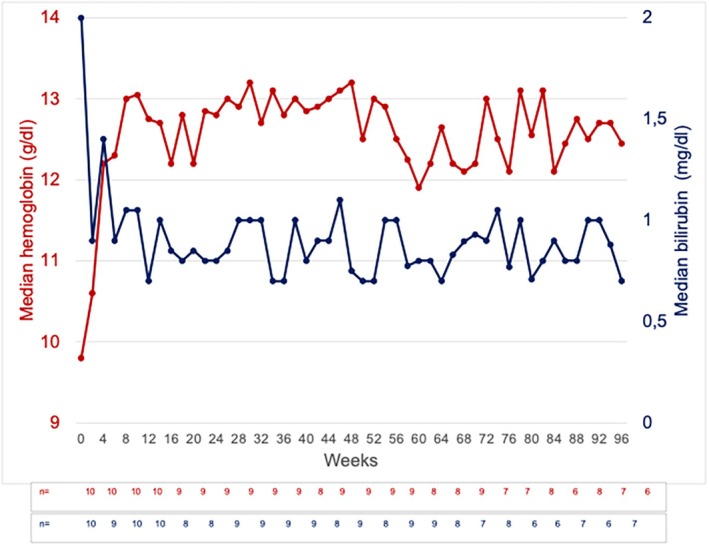
Median hemoglobin and bilirubin concentrations following sutimlimab treatment. Week 0 corresponds to baseline values, before the first dose of sutimlimab was applied. The red line represents hemoglobin and the blue line bilirubin concentrations. “*n*” indicates the number of patients assessed for hemoglobin (red) and bilirubin (blue).

**FIGURE 3:**
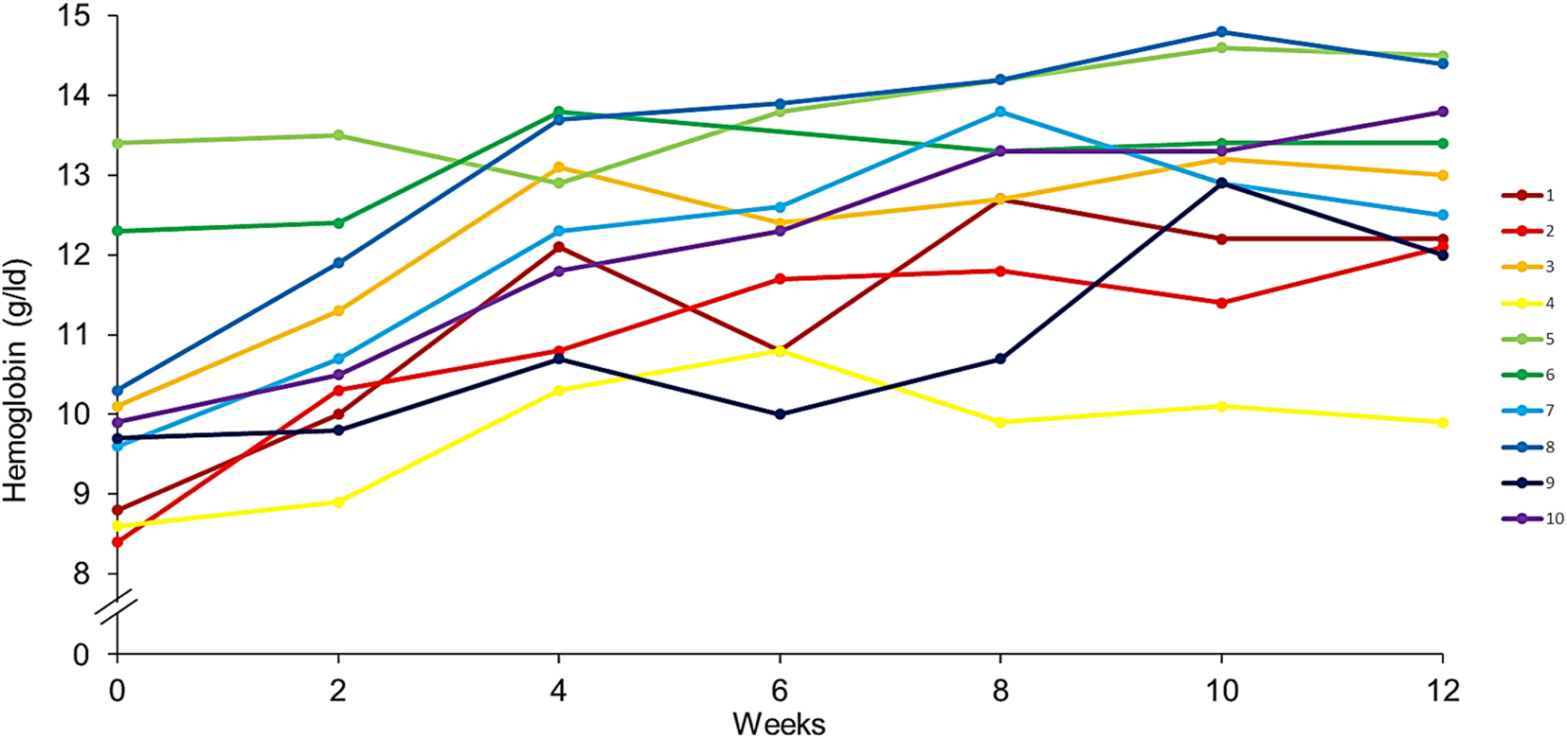
Hemoglobin concentrations during the first 12 weeks of therapy with sutimlimab. Week 0 corresponds to baseline values, before the first dose of sutimlimab was applied. The individual results of all 10 patients are given. Each color and line corresponds to one patient.

**FIGURE 4:**
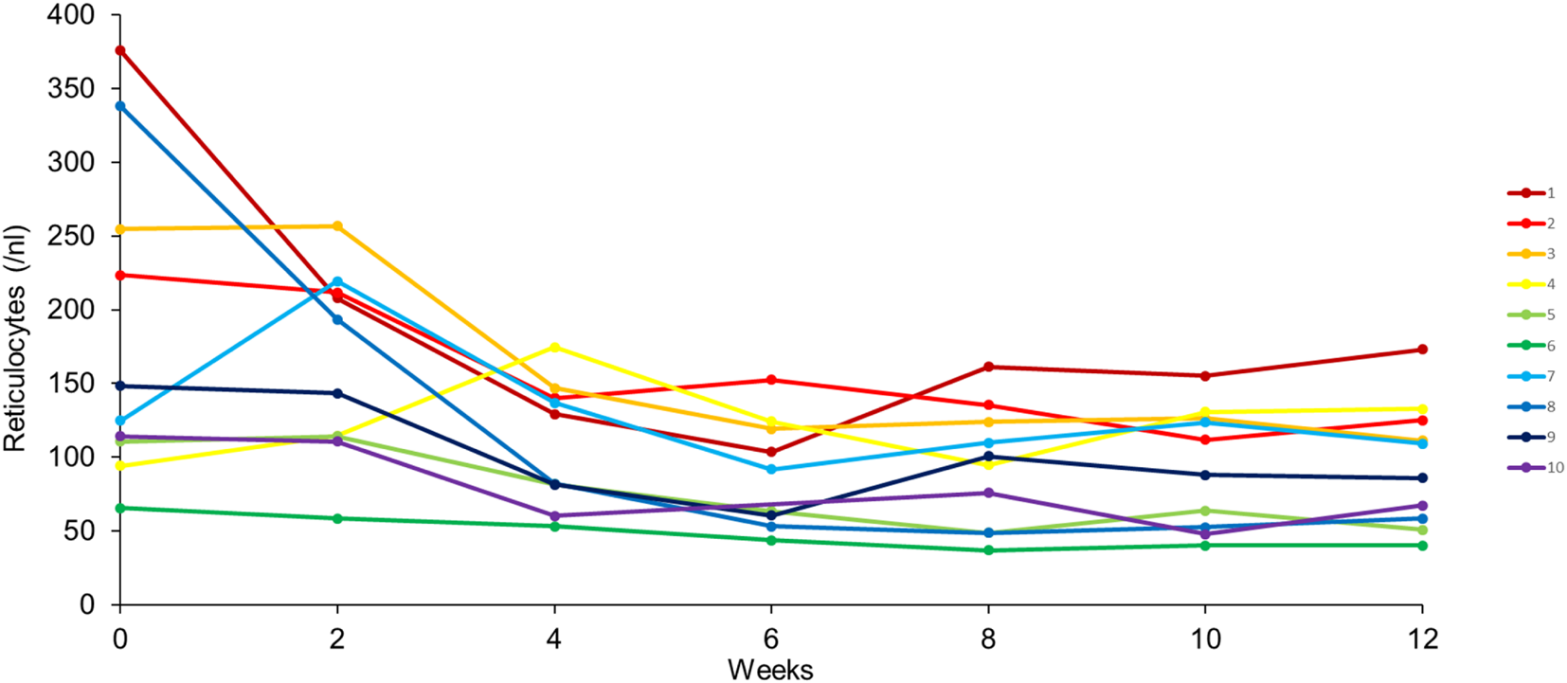
Reticulocyte count (Reference values 30–80 × 10^9^/L) as a function of time after treatment with sutimlimab. Week 0 corresponds to baseline values, before the first dose of sutimlimab was applied. Each color and line corresponds to one patient. When compared with the baseline, reticulocyte count decreased or remained stable at week two with the exception of a substantial increase in Patient No. 7.

Following treatment with sutimlimab, an immediate and sustained increase in hemoglobin concentration and a normalization of total bilirubin level were observed, indicating a rapid reduction of RBC destruction. The hemoglobin level increased in all patients except in one, with a pre‐therapy hemoglobin of 13.4 g/dL (Figure 3). This patient had initially presented with a hemoglobin of 8.3 g/dL at the beginning of the CADENZA trial. Six months later, her hemoglobin had stabilized above 12.4 g/dL and remained within the normal range even during the washout phase. Median hemoglobin concentrations increased from 9.8 g/dL (range 8.4–13.4 g/dL) at baseline to 10.6 g/dL (range 8.9–13.5 g/dL) at Week 2 and 12.2 g/dL (range 10.8–13.8 g/dL) at Week 4 and remained stable until the end of the study at Week 96 (median 12.5 g/dL, range 10.3–13.7 g/dL) (Figure 2). No patient needed blood transfusions during the observation period. A reduction in the median reticulocyte count from 136.5/nL (range 65.5–375.7/nL) at baseline to 76.7 nL (range 63.1–195.3/nL) at Week 96 was observed. The reduction of the reticulocytes was already present at Week 2 in nine of 10 patients with the exception of one patient exhibiting an increase from 124.7 to 219.3/nL (Figure 4, Patient No. 7). Total bilirubin levels were within the normal range at Week 2 and remained mostly below the upper limit of normal through Week 96 (Figure 2).

A normalization of hemopexin concentrations was observed in all 10 patients, whereas, haptoglobin normalized in six out of 10 patients. Hemopexin was measured in seven out of 10 patients at the start of sutimlimab therapy. Five patients were already within the normal range. Two patients had a hemopexin level below the normal range, but normalized during the trial. Hemopexin of the other three patients, not determined at the beginning of the MAP trial, but measured later on, showed a value within the normal range. The soluble transferrin receptor concentrations were assessed in three patients and the values were in the upper normal range. Inhibition of the classical complement pathway activity with sutimlimab resulted in normalization of complement protein C4 in all nine patients analyzed during therapy.

During therapy with sutimlimab, no new agglutination‐mediated circulatory CAD‐symptoms were observed. In general the treatment was well tolerated and there were no documented meningococcal or pneumococcal infections or further treatment‐related adverse events. One patient died after Week 16 due to complications of an underlying cardiovascular disease unrelated to sutimlimab.

## 4 Discussion

Primary CAD is an acquired mature B‐cell lymphoproliferative disease that lacks the MYD88 L265P mutation, displaying, however, recurrent trisomies of chromosomes 3, 12, and 18 and recurrent mutations in KMT2D and CARD11 [3]. Spontaneous remission of CAD is rather exceptional. Thus, long‐term management of CAD is often necessary to control hemolytic activity with all its consequences. In contrast to rituximab or rituximab plus bendamustine, sutimlimab has a more favorable safety profile because it does not cause myelosuppression, B‐cell depletion or hypogammaglobulinemia. This is clinically relevant because many patients with CAD are elderly and have additional comorbidities. As a corollary, monitoring long‐term therapeutic effects is of utmost importance. In the present investigation, we followed 10 patients with CAD on sutimlimab for 96 weeks in one single center after a median washout time of 12.6 weeks after participation in the CADENZA or CARDINAL trials. The analysis of these observations reveals significant efficacy and absence of relevant side effects in all patients.

The patients in the present study are representative of the primary CAD population. CAD typically occurs in individuals older than 50 years, with the greatest incidence in the seventh and eighth decades of life. The age of our patients with a median of 70.5 years and a range of 56–84 years was comparable with the age of the CAD population in general [13, 14]. In our group of patients analyzed, eight out of 10 were females. An overrepresentation of females in the CAD population has previously been noticed [14, 15].

Hemolytic anemia is the cardinal symptom in CAD patients. Therefore, many of these patients require transfusions at least at one point during the course of the disease [16]. An increase in hemoglobin levels and transfusion independency was demonstrated in most patients in the two large trials with sutimlimab (CARDINAL, CADENZA) [8, 15]. In the present investigation, a substantial increase of hemoglobin of 2.4 g/dL was observed from a median baseline of 9.8–12.2 g/dL within 4 weeks of sutimlimab therapy, a beneficial effect that persisted up to the 96‐week follow‐up (Figure 2). All patients remained transfusion‐independent throughout the treatment period with sutimlimab. The increase in hemoglobin was attributed to an abrogated hemolysis. The evidence for reduced hemolysis is severalfold: the decreased pre‐therapy level of hemopexin increased to the normal range in all patients, haptoglobin normalized in six out of 10 patients, bilirubin concentrations stabilized within the normal range during the 96 weeks of observation (Figure 2) and the reticulocyte count reached the normal range in nine out of 10 patients.

The Ii blood group was discovered when the “I” and “i” antigens were found to be crucial for the IgM binding to RBC in the CAD [17]. Since the I antigen is already present in the erythroid lineage, the reticulocytes also express the I antigen [18]. Thus, the cold agglutinin antibodies not only have the potential to bind to erythrocytes, but also to reticulocytes. Interestingly, Patient No. 7 had a pronounced increase in the reticulocyte count in the presence of an increase in hemoglobin, immediately after starting sutimlimab, whereas, in all the other patients the reticulocytes decreased or remained more or less stable, while their hemoglobin increased (Figures 3 and 4). One might hypothesize that cold agglutinin antibodies destroyed reticulocytes (reticulocyte lysis) before sutimlimab therapy was started in Patient No. 7, a hypothesis supported by a cold agglutinin antibody level above the upper detectable range measured at baseline in this patient.

Hemolysis leads to an increased production rate of bilirubin in CAD patients. Owing to the short half‐life of bilirubin, it represents a reliable marker for extravascular hemolysis and for monitoring the therapeutic response to sutimlimab. However, prolonged hyperbilirubinemia is associated with the formation of black pigment gallstones [19]. Cholecystitis has been observed in the CARDINAL trial, but interestingly only isolated cases of CAD and cholecystectomy have been published so far [15].

Complement‐mediated destruction of erythrocytes leads to an increase in extracellular hemoglobin due to hemolysis. The extracellular hemoglobin is then bound to haptoglobin. When hemolysis is pronounced and the capacity of haptoglobin to bind hemoglobin is saturated, free hemoglobin is cleaved into globin and heme. Heme is then bound to hemopexin, the protein with the highest affinity for heme [20, 21, 22]. As a consequence of hem‐binding, hemopexin levels decrease in the peripheral blood. Thus, hemopexin levels are inversely related to the severity of hemolysis. In all our patients on sutimlimab, the hemopexin levels were within the normal range during therapy, either from baseline or during treatment, indicating a beneficial effect of sutimlimab on hemolysis. Of note, normalization of haptoglobin, the most sensitive marker of hemolysis, was even more remarkable under complement inhibition.

Increased soluble transferrin receptor concentrations correlate positively with tissue iron deficiency or with stimulated erythropoiesis by hemolysis and/or by ineffective erythropoiesis [23, 24]. In our investigation, the soluble transferrin receptor concentration was randomly measured once in three patients during therapy. The values were in the normal range at Weeks 36, 70, and 80, respectively. These results indicate a steady state of erythropoiesis. Further investigations have to be performed to assess the utility of soluble transferrin receptor concentrations in patients with CAD.

Inhibition of the complement cascade increases the risk of infections with encapsulated bacteria [25]. Such infections are therefore of concern when sutimlimab is prescribed. However, sutimlimab is a first‐in‐class, humanized monoclonal antibody that specifically targets C1s inhibiting classical complement activation, while preserving the alternative and lectin pathways (Figure 1) [4, 26]. This mechanism of sutimlimab action may result in a reduced risk of infections with encapsulated bacteria in comparison with other complement inhibitors, particularly those targeting C5 [10]. The overall incidence of meningococcal infections in patients undergoing treatment with a C5 inhibitor has been reported to be as high as 0.25 per 100 patient‐years [27]. In line with the abovementioned pathophysiological explanation of sutimlimab action, no meningococcal infections have been observed in the CARDINAL and CADENZA studies. Although the risk of an increase in infections with encapsulated organisms appears theoretically to be minimal in patients given C1 inhibitor, vaccination against *Neisseria meningitidis* (including serogroup B meningococcus), *Haemophilus influenzae* , and *Streptococcus pneumoniae* is recommended in patients given sutimlimab [10]. Despite vaccination, severe infections occurred in several patients, with approximately half of these cases involving a disease with an encapsulated organism in the CARDINAL study [15]. A higher incidence of infections was observed, particularly in patients with risk factors for infections, such as aspiration, diabetes mellitus, prednisolone treatment, or urinary catheters [15]. In the placebo‐controlled CADENZA study, the infection rate for the sutimlimab arm was found to be only minimally increased in comparison to the placebo group [8]. Similarly, no meningococcal infection or other serious infections were observed during the entire study in our 10 patients, which might be best explained by the absence of risk factors for infections or other comorbidities and vaccination. Since CAD patients have to be treated lifelong, booster vaccination after 3–6 years as recommended in immunocompromised patients has to be applied [28]. Furthermore, patients should be provided with a stand‐by antibiotic (e.g., amoxicillin/clavulanic acid 1000 mg) in case of suspected meningococcal or other severe infection. The complement system is relevant not only for encapsulated organisms but also for the elimination of various pathogens and, additionally, for the development, differentiation and local homeostasis of other cellular functions [29]. Therefore, further long‐term observations are required for a more comprehensive understanding of potential side effects associated with sutimlimab therapy.

Sutimlimab is a humanized immunoglobulin G4 (IgG4) monoclonal antibody [8]. As it is the case with any xenobiotic, an antidrug antibody (ADA) may be pre‐existing or induced following the administration of modified human antibodies. These ADAs could reduce or abrogate the therapeutic effect of sutimlimab. Fortunately, such ADAs have very rarely been described for sutimlimab in the past [15]. Since no evidence of a resistance or failure of sutimlimab was encountered in the present study, the ADAs were not determined in our patients. Throughout the study, there was no clinical or laboratory evidence of drug‐induced autoimmune hemolysis.

The results of a similar study conducted in Japan by Miyakawa et al. (hereafter referred to as the OLE trial) have recently been reported [30]. Both, the group of patients in the OLE trial from Japan and the MAP trial from Essen, participated in either the CADENZA or the CARDINAL study [8, 12], before entering these two long‐term extension studies. Comparing the OLE and MAP trials reveals virtual identity with respect to median age, overrepresentation of women and sutimlimab dose. In addition, all patients were vaccinated as required in the CADENZA and CARDINAL trials [8, 12]. However, the extension studies were complementary or differed with respect to several parameters. The subjects enrolled in the OLE trial were seven Asian patients treated in different medical centers, whereas, the 10 patients in the MAP trial were Caucasians followed in a single center. The duration of the OLE trial was 47 weeks after a washout period of 70 days between the CARDINAL/CADENZA investigations and the start of the OLE study, whereas the follow‐up in the MAP trial was 96 weeks with a comparable washout period. Hemolysis parameters appeared to be slightly more pronounced at baseline in the OLE study, than in the MAP trial (median hemoglobin 8.8 vs. 9.8 g/dL and median bilirubin 2.7 vs. 2 mg/dL). With regard to the therapeutic endpoint, no blood transfusions were required in both study groups, and the median hemoglobin concentration increased in both groups; also the increase assessed at Week 46 was marginally more distinct in the MAP group (13.1 g/dL, range 10.8–13.8 g/dL) than in the OLE study (11.3 g/dL, range 7.6–14.6 g/dL). These beneficial effects of sutimlimab were maintained through Week 96 in the patients from the MAP study (12.5 g/dL, range 10.3–13.7 g/dL). The bilirubin kinetics were similar in both groups with by and large normalization in all patients. One patient died in each group. The cause of death was not considered by the investigators to be a treatment‐related serious event due to sutimlimab. Besides the focus on the parameters of pivotal relevance, hemoglobin and bilirubin, the investigators of the MAP trial also attempted to establish the effect of sutimlimab therapy on reticulocytes, haptoglobin, hemopexin, soluble transferrin receptor and complement C4.

## 5 Limitations and Conclusion

The present small single center study confirms the efficacy and acceptable rate of side effects of sutimlimab in CAD patients. The total observation time with sutimlimab was up to 5.25 years (63 months) including a several weeks therapy‐free washout phase with the reappearance of the disease state. Although our investigation did not comprise a control group, it strongly backs the conclusion that sutimlimab can be recommended as a first‐line therapy for symptomatic hemolytic CAD patients.

## Author Contributions

S.M.F. contributed to data analysis, performed the data interpretation, and drafted the manuscript. P.H. collected and analyzed the data. F.A. contributed to data collection and data analysis. V.L. and H.C.R. provided critical revision of the manuscript for important intellectual content. A.R. designed the study, contributed to data interpretation, and provided critical revision of the manuscript for important intellectual content. All authors approved the final version of the manuscript.

## Funding

The authors have nothing to report.

## Ethics Statement

This study was performed in accordance with the study protocol, the Declaration of Helsinki, and the International Council for Harmonisation guidelines for Good Clinical Practice.

## Consent

All patients provided written informed consent for their participation in the study.

## Conflicts of Interest

F.A. reports consultancy for Bristol Myers Squibb/Celgene, Global Blood Therapeutics/Pfizer, Novartis, and Vertex; honoraria from Agios, Bristol Myers Squibb/Celgene, Global Blood Therapeutics/Pfizer, Novartis, and Vertex; and research funding from Global Blood Therapeutics/Pfizer. H.C.R. reports consultancy for Roche, Vertex, Janssen, and KinSea; honoraria from Abbvie, Aurikamed, Roche, Novartis, Takeda, and Amgen; research funding from Gilead and AstraZeneca; ownership interests in Fresenius, Roche, Novartis, Lilly, and Janssen; and is the founder of CDL Therapeutics GmbH. A.R. has received consultancy fees from Alexion Pharmaceuticals Inc., Apellis Pharmaceuticals, Bioverativ, a Sanofi company, Novartis, Roche, and Sanofi; honoraria from Alexion, Amgen, Apellis, Novartis, Roche, Sanofi and Sobi, and advisory board fees from Alexion, Amgen, Apellis, Bioverativ, Novartis, Roche, Sanofi, and Sobi. The other authors declare no conflicts of interest.

## Data Availability

The data that support the findings of this study are available from the corresponding author upon reasonable request.
